# A non-invasive specimen collection method and a novel simian foamy virus (SFV) DNA quantification assay in New World primates reveal aspects of tissue tropism and improved SFV detection

**DOI:** 10.1371/journal.pone.0184251

**Published:** 2017-09-01

**Authors:** Cláudia P. Muniz, HaoQiang Zheng, Hongwei Jia, Liliane T. F. Cavalcante, Anderson M. Augusto, Luiz P. Fedullo, Alcides Pissinatti, Marcelo A. Soares, William M. Switzer, André F. Santos

**Affiliations:** 1 Departamento de Genética, Universidade Federal do Rio de Janeiro, Rio de Janeiro, Brazil; 2 Centers for Disease Control and Prevention, Atlanta, Georgia, United States of America; 3 Fundação Jardim Zoológico da Cidade do Rio de Janeiro, Rio de Janeiro, Brazil; 4 Centro de Primatologia do Rio de Janeiro, Instituto Estadual de Ambiente, Rio de Janeiro, Brazil; 5 Programa de Oncovirologia, Instituto Nacional de Câncer, Rio de Janeiro, Brazil; University of Pittsburgh Centre for Vaccine Research, UNITED STATES

## Abstract

Simian foamy viruses (SFVs) co-evolved with a wide range of Old World and New World primates (OWPs and NWPs, respectively) and occasionally transmit to humans. Previous studies of OWPs showed that the predominant site of SFV replication is the oral mucosa. However, very little is known about SFV viral loads (VLs) in the oral mucosa or blood of NWPs. NWPs have smaller body sizes, limiting collection of sufficient whole blood volumes to molecularly detect and quantify SFV. Our study evaluated the use of noninvasively collected buccal swabs to detect NWP SFV compared with detection in blood using a new NWP SFV quantitative PCR (qPCR) assay. Buccal and blood samples were collected from 107 captive NWPs in Brazil comprising eleven distinct genera at the Primate Center of Rio de Janeiro (n = 58) and at Fundação Jardim Zoológico da Cidade do Rio Janeiro (n = 49). NWP SFV western blot (WB) testing was performed on a subset of animals for comparison with PCR results. The qPCR assay was validated using distinct SFV polymerase sequences from seven NWP genera (*Callithrix*, *Sapajus*, *Saimiri*, *Ateles*, *Alouatta*, *Cacajao* and *Pithecia*). Assay sensitivity was 20 copies/10^6^ cells, detectable in 90% of replicates. SFV DNA VLs were higher in buccal swabs (5 log copies/10^6^ cells) compared to peripheral blood mononuclear cells (PBMCs) (3 log copies/10^6^ cells). The qPCR assay was also more sensitive than nested PCR for detection of NWP SFV infection and identified an additional 27 SFV-infected monkeys of which 18 (90%) were WB-positive and three that were WB-negative. We show the utility of using both blood and buccal swabs and our new qPCR assay for detection and quantification of diverse NWP SFV, which will assist a better understanding of the epidemiology of SFV in NWPs and any potential zoonotic infection risk for humans exposed to NWPs.

## Introduction

Foamy viruses (FVs) are complex retroviruses identified in different mammalian orders [1]. Simian FVs (SFVs) infect many nonhuman primates (NHPs), including Old World primates (OWPs) [2–7], which can also infect humans zoonotically [8–10]. In contrast, little is known about SFV infection in New World primates (NWPs) and their ability to infect humans. We have previously described the first molecular prevalence of SFV in the wild and in captive neotropical primates and reported the presence of SFV in 14 of 16 NWPs genera analyzed, with prevalence varying between 14–30% [11]. More recently, Ghersi *et al*. developed and validated specific ELISA and western blot (WB) tests as well as generic PCR assays to assess the prevalence of SFV in NWPs living in U.S. zoos and at rescue centers and trade markets in Peru [12]. Of the captive NWPs from the U.S., 45% were SFV WB-positive, while 38% of captive and wild-caught NWPs from Peru were positive [12]. Following this study, we used the same serologic and PCR methods to investigate the prevalence and species distribution of NWP SFV in 140 captive monkeys from Brazil, finding an overall serologic and molecular prevalence of 43% and 34%, respectively, and identified distinct SFV in four NWP genera [13]. Although a high prevalence and wide diversity of NWP SFV exists, only a single study examined the risk of SFV infection in workers exposed to NWP [14]. That study showed serologic evidence of NWP SFV infection for eight persons, however PCR testing was negative [14].

Specimens collected by invasive methods, mostly venipuncture, are commonly used to detect SFV, including detection of antibodies in plasma [15], viral load (VL) using genomic DNA (gDNA) from peripheral blood mononuclear cells (PBMC) by PCR [11, 16] and/or co-culture of infected PBMC with susceptible cell lines [17]. The two latter methods can be challenging for NWPs because limited volumes of blood can be collected due to their small physical size. Moreover, invasive methods may risk the animal’s health and some NWPs are endangered species, further restricting those approaches. An alternative, noninvasive method was used for SFV detection by PCR in fecal samples of wild NHPs and in captive OWPs sharing the same enclosure [18]. However, this technique requires the development of species-specific microsatellite methods for individual identification, a difficult task given the greater diversity of NWPs with over 110 distinct species described to date [19]. Combined with reports of SFV replication in the oral mucosa [20] and saliva of infected humans and OWPs [16, 21–23], we evaluated the use of noninvasively collected buccal swabs to detect and quantify NWP SFV compared with detection in matching blood specimens. For this comparison, we developed novel quantitative PCR (qPCR) assays specific for NWP SFV and host sequences.

## Results

### Detection of SFV DNA in blood and buccal swab samples by nested PCR

Buccal swabs (n = 30) and PBMC specimens (n = 97) were collected from 107 NWPs housed at the Primate Center of Rio de Janeiro (CPRJ) (n = 58) and at the Fundação Jardim Zoológico da Cidade do Rio Janeiro (RIOZOO) (n = 49). For 27 monkeys both PBMC and buccal swabs were collected; three monkeys only had buccal swabs collected. Intact PBMC gDNA, as determined by PCR detection of cytochrome B (*cytB*) mitochondrial DNA (mtDNA) sequences, was present in all 97 NWPs with that specimen type. gDNA was also present in all 30 buccal swab specimens. However, the amount of gDNA from each specimen was not sufficient for testing with all PCR assays (Table 1).

**Table 1:** Simian foamy virus (SFV) Western blot (WB), nested PCR^1^, and quantitative PCR (qPCR) results and range of SFV DNA viral load in peripheral blood mononuclear cells (PBMCs) and buccal swab samples in New World primates from Brazil.

Primate species	WB	PBMCs			Buccal swab		
		Nested PCR	qPCR	Log10 range SFV copies/10^6^ cells	Nested PCR	qPCR	Log10 range SFV copies/ 10^6^ cells
*Alouatta belzebul* (n = 1)	100% (1/1)	100% (1/1)	100% (1/1)	4.53	0% (0/1)	ND	ND
*Alouatta fusca clamitans* (n = 2)	100% (2/2)	100% (2/2)	100% (2/2)	1.98–3.4	100% (1/1)	ND	ND
*Alouatta guariba* (n = 2)	100% (2/2)	100% (2/2)	50% (1/2)	Undetectable—1.8	0% (0/2)	ND	ND
*Alouatta guariba clamitans* (n = 1)	100% (1/1)	100% (1/1)	100% (1/1)	2.78	ND^2^	ND	ND
*Alouatta seniculus* (n = 1)	100% (1/1)	100% (1/1)	100% (1/1)	1.28	0% (0/1)	100% (1/1)	5.04
*Aotus nigriceps* (n = 1)	ND	0% (0/1)	100% (1/1)	2.62	0% (0/1)	100% (1/1)	4.87
*Aotus sp* (n = 3)	50% (1/2)	33.3% (1/3)	100% (3/3)	2.6–4	ND	ND	ND
*Ateles paniscus* (n = 1)	ND	0% (0/1)	0% (0/1)	Undetectable	ND	ND	ND
*Brachyteles arachnoides* (n = 2)	ND	0% (0/1)	0% (0/1)	Undetectable	100% (1/1)	100% (1/1)	7.29
*Cacajao melanocephalus* (n = 3)	100% (2/2)	66.6% (2/3)	66.6% (2/3)	3.3–5.69	0% (0/2)	100% (2/2)	5.33–5.37
*Callimico sp* (n = 1)	ND	100% (1/1)	100% (1/1)	4.6	ND	ND	ND
*Callithrix aurita* (n = 2)	100% (1/1)	100% (2/2)	100% (2/2)	2.1–2.36	0% (0/1)	0% (0/1)	ND
*Callithrix jacchus (n = 1)*	ND	0% (0/1)	ND	ND	ND	100% (1/1)	5.64
*Callithrix geoffroyi* (n = 1)	100% (1/1)	100% (1/1)	ND	ND	100% (1/1)	100% (1/1)	2.59
*Cebus olivaceus* (n = 1)	ND	100% (1/1)	100% (1/1)	4.24	ND	ND	ND
*Chiropotes sp* (n = 1)	100% (1/1)	100% (1/1)	0% (0/1)	ND	ND	ND	ND
*Leontopithecus chrysomelas* (n = 15)	75% (3/4)	28.6% (4/14)	57.1% (8/14)	1.79–3.9	37.5% (3/8)	80% (4/5)	2.9–7.3
*Leontopithecus chrysopygus* (n = 8)	ND	0% (0/8)	25% (2/8)	2.32–2.77	ND	ND	ND
*Leontopithecus rosalia* (n = 3)	100% (2/2)	66.6% (2/3)	66.6% (2/3)	1.51–2.69	100% (1/1)	100% (1/1)	5.46
*Saguinus bicolor* (n = 1)	ND	0% (0/1)	100% (1/1)	2.42	ND	ND	ND
*Saimiri sciureus* (n = 1)	ND	100% (1/1)	100% (1/1)	2.17	100% (1/1)	100% (1/1)	3.3
*Saimiri ustus* (n = 7)	100% (5/5)	20% (1/5)	66.6% (2/3)	1.94–2.09	100% (4/4)	100% (7/7)	2.15–6.36
*Sapajus apella* (n = 14)	100% (13/13)	35.7% (5/14)	83.3% (9/12)	1.45–4.96	ND	ND	ND
*Sapajus flavius* (n = 1)	0% (0/1)	100% (1/1)	100% (1/1)	4.13	ND	ND	ND
*Sapajus robustus* (n = 9)	85.7% (6/7)	44.4% (4/9)	62.5% (4/8)	1.68–4.51	ND	100% (1/1)	3.37
*Sapajus sp*. (n = 7)	100% (7/7)	0% (0/1)	57.1% (3/7)	1.25–3.67	ND	ND	ND
*Sapajus xanthosternos* (n = 17)	100% (14/14)	64.7% (11/17)	87.5% (13/16)	1.74–4.8	40% (2/5)	100% (2/2)	3.59–6.67
Total (n = 107)	94% (63/67)	46.4% (45/97)	65.3% (62/95)	Undetectable– 5.69	46.7% (14/30)	92% (23/25)	2.15–7.3

^1^ Postivity by nested PCR includes a specimen testing positive in at least one of three nested PCR assays for polymerase (192-bp or 520-bp) or LTR-gag (398-bp) sequences.

^2^ ND, not done, indicating the test was not performed and/or specimen quantity was insufficient for testing or the specimen was not collected.

To detect SFV DNA in NWPs, we first performed a screening nested PCR for short polymerase (*pol*) sequences (192-bp) using generic primers and PBMC gDNA as previously described [11]. In addition, generic primers were also used with PBMC gDNA to amplify two additional SFV genomic regions, a 398-bp LTR/*gag*-sequence (225-bp in LTR and 173-bp in *gag*) and a 520-bp *pol* fragment, using nested PCR [11]. Table 1 shows the nested PCR results for the PBMC and buccal swab samples for the 27 NWP species examined in our study. SFV sequences were detected in the majority of each NWP species. Of the 97 PBMC specimens, 46.4% (45/97) tested positive for at least one SFV region by nested PCR and included 39.3% (22/56) of animals in CPRJ and 56.1% (23/41) at RIOZOO. Of the 30 buccal swab samples, 46.7% (14/30) tested positive for at least one SFV sequence by nested PCR. From CPRJ, 43.8% (7/16) were SFV-positive, while 50% (7/14) tested SFV-positive using buccal gDNA from monkeys at RIOZOO.

Of the 45 SFV-positive animals in the PBMC compartment by nested PCR, 19(42.2%) had a buccal swab collected, and 9 (45%) of those were PCR-positive for at least one SFV sequence by nested PCR in that compartment. Of 52 SFV-negative animals by nested PCR of PBMC gDNA, eight (15.4%) had oral swabs collected and three (37.5%) were PCR-positive in that compartment. Two of three monkeys with only buccal swab specimens were tested by nested PCR and both were positive. The third monkey was only tested by qPCR (see below for results).

### Real-time PCR assay performance

To simultaneously detect infection with NWP SFV and quantify the amount of virus present in the specimens we developed a new qPCR assay to generically detect divergent SFV *pol* sequences. This diagnostic SFV *pol* qPCR assay successfully amplified *pol* sequences of all seven divergent NWP SFV strains from *Callithrix jacchus* (SFVcja), *Sapajus xanthosternos* (SFVsxa), SFVssp (*Saimiri* species), *Ateles* species (SFVasp), *Alouatta guariba* (SFVagu), *Cacajao melanocephalus* (SFVcme), and *Pithecia* species (SFVpsp), comprising viral strains from each NWM family (*Atelidae*, *Cebidae*, and *Pitheciidae*) (data not shown). Assay linearity ranged from 10^7^ to one copy of each plasmid. The qPCR test could detect 100% of all replicates containing 100 and 50 copies for all SFV strains (data not shown) and 20 copies of SFVcme in 90% of replicates and 100% of replicates for all six remaining SFV strains, giving an overall assay sensitivity of 20 NWP SFV copies/reaction (Table 2). Assay sensitivity for all seven SFV strains decreased below 20 copies/reaction (Table 2). Specificity of the assay was 100% using gDNA from 35 U.S. blood donors and from 30 SFV WB-negative and PCR-negative NWPs.

**Table 2:** Sensitivity of the qPCR assay specific to New World primate simian foamy virus (SFV)^1^.

	Sensitivity				
SFV strain^2^	20 copies	10 copies	5 copies	3 copies	1 copy
SFVasp	100% (10/10)	70% (7/10)	80% (8/10)	50% (5/10)	40% (4/10)
SFVcja	100% (10/10)	90% (9/10)	70% (7/10)	70% (7/10)	30% (3/10)
SFVsxa	100% (10/10)	70% (7/10)	60% (6/10)	70% (7/10)	60% (6/10)
SFVagu	100% (10/10)	100% (10/10)	100% (10/10)	80% (8/10)	30% (3/10)
SFVcme	90% (9/10)	90% (9/10)	80% (8/10)	50% (5/10)	10% (1/10)
SFVpsp	100% (10/10)	100% (10/10)	100% (10/10)	100% (10/10)	30% (3/10)
SFVssp	100% (10/10)	70% (7/10)	60% (6/10)	70% (7/10)	60% (6/10)

1. Number of positive reactions/number of replicates tested in parentheses.

2. SFV strain codes: SFVasp (*Ateles* species), SFVcja (*Callithrix jacchus*), SFVsxa (Sapajus *xanthosternos*), SFVagu (*Alouatta guariba*), SFVcme (*Cacajao melanocephalus*), SFVpsp (*Pithecia* species), and SFVssp (*Saimiri* species).

To quantify the number of cells per reaction and normalize the SFV DNA VLs across specimens, a generic real-time qPCR for the primate *RPP30* (ribonuclease P/MRP, 30kDa subunit) gene was developed. The *RPP30* qPCR assay linear range was 10^7^—one copy and could detect 100% (20/20) and 65% of all replicates containing ≥ five and one copies, respectively, for an overall sensitivity of five *RPP30* copies/reaction (data not shown). The generic *RPP30* assay successfully detected *RPP30* sequences in all NWP samples used in the study.

### SFV DNA VL quantification in PBMCs

PBMC gDNA from 95 NWP was analyzed with the validated qPCR test. Sixty-five percent (62/95,) of the PBMC gDNAs had detectable levels of SFV at our cutoff of 20 copies/reaction, including detection of SFV in ten different NWP genera (Table 1), eleven genera if the *Ateles* plasmid control is included in the total. Normalized by using the *RPP30* qPCR assay, the average PBMC DNA VL of the 62 animals was 2.9 log SFV copies/10^6^ cells (standard deviation (SD) = 1.11 log SFV copies/10^6^ cells) and ranged from 1.25–5.60 log SFV copies/10^6^ cells.

Previous studies have shown that NHP-to-NHP transmission of SFV is associated with aggressive behavior as an animal becomes a juvenile [5]. Retroviral transmission has also been associated with higher viral loads. In order to evaluate potential correlations between age and transmission and SFV DNA VLs in NWP PBMC and buccal swabs, we classified all animals as either sexually mature or immature following zoological classification and considering specific sexual maturity characteristics of each species [24] (S1 Table). To evaluate possible DNA VL differences between mature and immature animals, we calculated the median SFV DNA VL in PBMCs of both groups (Table 3). Mature animals (n = 52) had an average DNA VL of 2.72 log SFV copies/10^6^ cells, compared to immature monkeys (n = 14) which had an average of 3.41 log SFV copies/10^6^ cells (p = 0.915). Sixty-seven percent (52/78) of the mature animals had detectable DNA VL in PBMC, compared to 82% (14/17) of the immature animals, a difference that was not significant (p = 0.255; Fisher’s exact test). For the *Sapajus* and *Leontopithecus* genera, enough PBMC specimens with detectable DNA VL and age data were available for within-genus comparisons. The proportion of mature animals was 100% (12/12) among the *Leontopithecus* animals (age range between 20.4 and 259.6 months) and of 66% (21/32) among the *Sapajus* monkeys (age range between 84 and 235.2 months). We then estimated the Pearson’s correlation coefficient between age and SFV DNA VL. The correlation coefficient for *Sapajus* was low (r = 0.02, p = 0.9), as well as for *Leontophithecus* (r = -0.11, p = 0.73), indicating an absence of correlation between animal age and DNA VL in these two species.

**Table 3:** Simian foamy virus (SFV) viral load (VL) in genomic DNA of 1 x 10^6^ cells from PBMC and buccal swabs in sexually mature and immature New World primates^1^.

	PBMC (n = 66)		Buccal swab (n = 23)	
Average **VL**	2.9 log		4.7 log	
Standard deviation **VL**	1.11 log		1.45 log	
Student’s *t* test	p < 0.0001			
Sexual maturity status	Mature (n = 52)	Immature (n = 14)	Mature (n = 17)	Immature (n = 6)
Average **VL**	2.72 log	3.41 log	4.9 log	4.64 log
Standard deviation **VL**	1.05 log	1.17 log	1.37 log	1.76 log
Student´s *t* test	p = 0.915		p = 0.9	

1. VL reported in log10 (SFV DNA copies/1 million cells)

### SFV DNA VL quantification in buccal swabs

Twenty-five NWP buccal swab gDNA samples were analyzed in our real-time qPCR assay, of which 23 (92%) had detectable SFV DNA VLs (Table 1). After normalizing for *RPP30* cell equivalents, the average DNA VL in buccal swabs for these 23 animals was 4.7 log SFV copies/10^6^ cells (SD = 1.45 log SFV copies/10^6^ cells) and ranged from 2.15–7.3 log SFV copies/10^6^ cells. No differences were found between the SFV DNA VLs in buccal swabs of sexually mature (n = 17) and immature (n = 6) animals (average of 4.9 log and 4.64 log copies/10^6^ cells, respectively; p = 0.9; Table 2). Ninety-four percent (17/18) of the mature animals had detectable DNA VL in the buccal swab, compared to 86% (6/7) of the immature animals, but this difference was not significant (p = 0.49; Fisher’s exact test).

### Comparison of SFV DNA VL in PBMCs and buccal swabs

**Fig 1:**
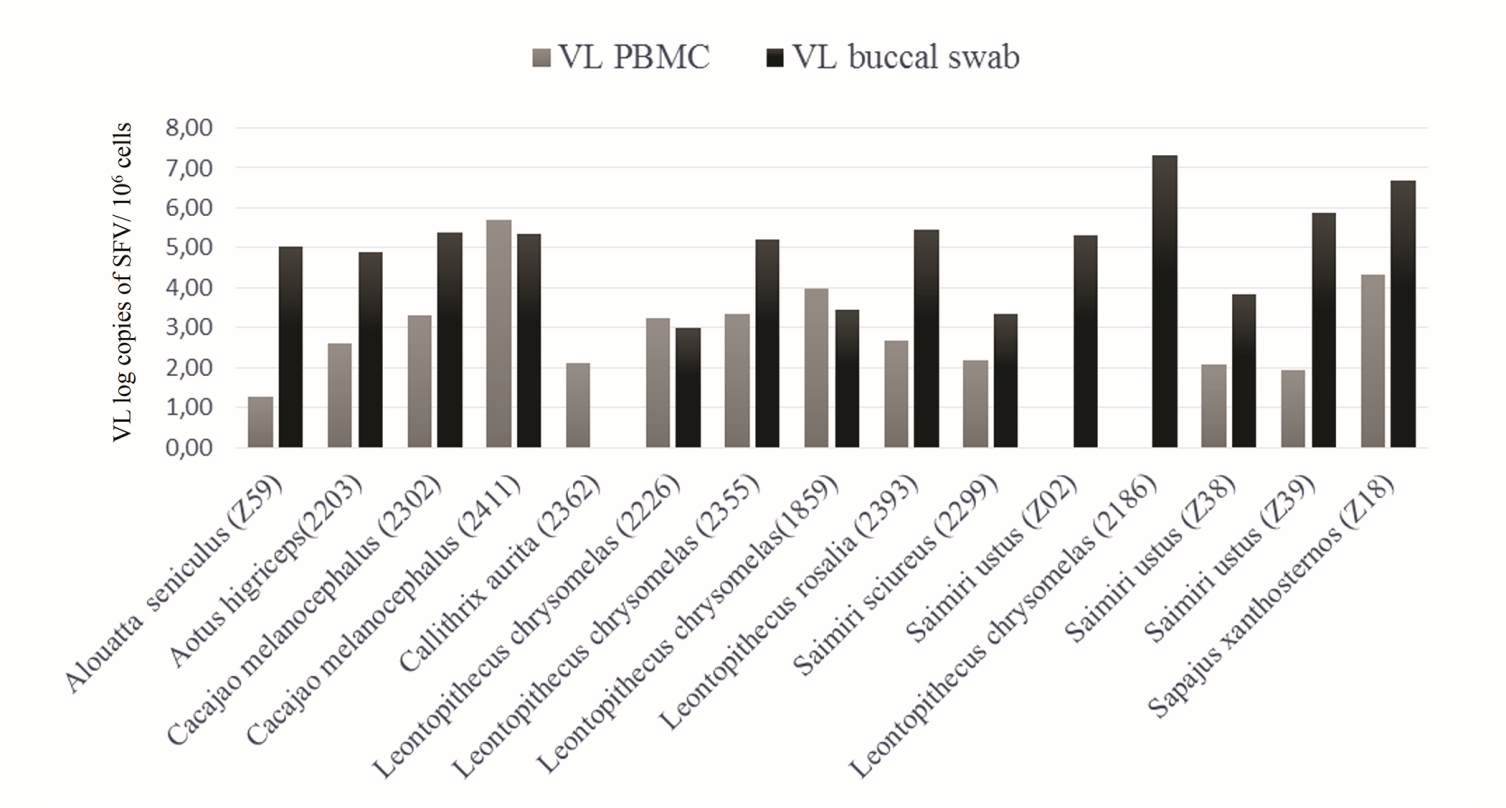
Simian foamy virus (SFV) DNA viral load (VL) comparison in PBMCs and buccal swabs for monkeys with both specimen types. Specimens from all 15 monkeys were tested in both body compartments by quantitative PCR. Individual monkey identification codes are in parentheses.

We observed a significant difference in SFV DNA VLs in PBMCs (n = 62, average of 2.9 log SFV copies/10^6^ cells) and in buccal swabs (n = 23, average of 4.7 log SFV copies/10^6^ cells) (p < 0.0001; Table 3). Among animals with detectable SFV DNA VLs in both PBMC and buccal swab specimens, buccal swab VLs were on average 1.7 log copies/10^6^ cells higher than those found in PBMCs. DNA VLs in matching PBMCs and buccal swabs were available for 15 different NWPs (Fig 1). Of those, a concordance in detectable VL in both compartments reached 80% (12/15), with only two animals for which VL was detectable in buccal swabs but undetectable in the PBMC, and one for which the opposite result was observed. The average SFV DNA VLs in these animals was 2.58 log SFV copies/10^6^ cells in PBMC and 4.67 log SFV copies/10^6^ cells in buccal swabs (p = 0.005). Buccal swab DNA VLs were up to 3.9 log higher in comparison with PBMCs (Table 1). However, we did not find a correlation between the SFV DNA VL in buccal swabs and PBMCs in these animals (r = - 0.07; p = 0.8). Of these 15 animals, nine were nested PCR-positive (60%) using PBMC DNA and 13 had enough buccal swab gDNA for testing of which five (38.5%) were nested PCR-positive.

### Comparison of qPCR with nested PCR results

Of 45 NWPs positive by nested PCR for at least one viral genomic fragment (diagnostic *pol*, longer *pol*, and/or *gag*/LTR) amplified from PBMCs, 39 were also tested by qPCR for SFV detection and DNA VL quantification. DNA VLs were detectable in 35 (89.7%) broadly diverse NWPs with an average of 3.21 log SFV copies/10^6^ cells (range 1.28–5.69 log SFV copies/10^6^ cells) (Table 1). These qPCR-positive NWPs included *Leontopithecus chrysomelas* (n = 4), *L*. *rosalia* (n = 1), *Sapajus xanthosternos* (n = 10), *Sapajus apella* (n = 3), *Sapajus robustus* (n = 2), *Sapajus sp*. (n = 1), *Sapajus flavius* (n = 1), *Saimiri sciureus* (n = 1), *Aotus* sp. (n = 1), *Alouatta belzebul* (n = 1), *Alouatta fusca clamitans* (n = 2), *Alouatta guariba clamitans* (n = 1), *Alouatta seniculus* (n = 1), *Callimico* sp. (n = 1), *Callithrix aurita* (n = 2), *Cebus olivaceus* (n = 1), and *Cacajao melanocephalus*(n = 2). Plasma was available for 25/35 qPCR-positive monkeys of which 24 (96%) were WB-positive for SFV antibodies. One immature *S*. *flavius* tested WB-negative which couls reflect a new infection.

In contrast, of 50 NWPs with negative PBMC results by nested PCR, 27 (54%) had detectable DNA VL with an average of 2.6 log SFV copies/10^6^ cells and a range of 1.26–4.46 copies SFV/10^6^ cells). These nested PCR-negative but qPCR-positive NWPs included *Alouatta guariba* (n = 1), *Aotus nigriceps* (n = 1), Aotus sp. (n = 2), *Leontopithecus chrysomelas* (n = 4), *L*. *chrysopygus* (n = 2), *L*. *rosalia* (n = 1), *Saguinus bicolor* (n = 1), *Saimiri ustus* (n = 2), *Sapajus apella* (n = 7), *Sapajus robustus* (n = 3), and *Sapajus xanthosternos* (n = 3). Twenty of these 27 NWPs had plasma available, and 18 (90%) were also WB-positive for antibodies to SFV Gag proteins. Specimens from one each mature *Aotus* sp. and *L*. *chrysomelas* were WB-negative but qPCR-positive and may represent recent infections.

Of the 14 NWP buccal swabs positive by nested PCR for at least one viral fragment, gDNA was available for DNA VL quantification from 10 monkeys and all 10 (100%) had detectable DNA VL (average of 5.13 log SFV copies/10^6^ cells; range 2.59–7.29 log SFV copies/10^6^ cells). For 16 nested PCR-negative NWP buccal swabs, gDNA was available for nine samples for qPCR testing of which seven (78%) had detectable DNA VL (average of 5.23 log SFV copies/10^6^ cells; range 3.45–7.30 SFV copies/10^6^ cells). These seven primates included *L*. *chrysomelas* (n = 3), *Cacajao melanocephalus* (n = 2), *Aotus nigriceps* (n = 1) and *Alouatta seniculus* (n = 1). Plasma samples were available for WB testing for four of these seven animals with negative nested PCR but detectable DNA VL results, of which three were positive, including two *Cacajao melanocephalus* and one *Alouatta seniculus*.

We also identified nine monkeys from four genera (*S*. *robustus* (n = 1), *Sapajas* species (n = 3), *S*. *apella* (n = 2), *A*. *guariba* (n = 1), *L*. *rosalia* (n = 1), and *Chiropotes* species (n = 1)) that all tested WB-positive but gave undetectable PBMC qPCR results. Of these, PBMC gDNA from four tested positive by nested PCR, including one each of *S*. *robustus*, *L*. *rosalia*, *A*. *guariba*, and *Chiropotes* species. Buccal gDNA was only available for one (*A*. *guariba*) of these nine animals and it was nested PCR-negative but positive by nested PCR testing of PBMC gDNA. Of four WB-negative animals (one each *L*. *chrysomelas*, *S*. *robustus*, *Aotus* species, and *S*. *flavius*), three were qPCR-positive using PBMC gDNA (*S*. *robustus*, *Aotus* species, and *S*. *flavius*) of which one was also nested PCR-positive (*S*. *flavius*). Buccal gDNA was available for only the *L*. *chrysomelas* which was qPCR-positive but nested PCR-negative.

A higher proportion of qPCR-positive samples were present among PBMC samples testing positive by nested PCR-positive (35/39, 89.7%) compared to nested PCR-negative PBMC samples (27/50, 54%) (p = 0.0003). For buccal specimens there was no significant difference in qPCR-positive, nested PCR-positive animals (10/10, 100%) and qPCR-positive, nested PCR-negative animals (7/9 = 77.7%) (p = 0.1243). Analysis of sexual status, primate species, and gender did not find a difference in qPCR and nested PCR results in immature or mature animals or in males versus females or by primate species. To determine if low VL impacted the ability to detect SFV infection using nested PCR we statistically compared SFV DNA VLs with negative and positive nested PCR results in both specimen compartments. We found that lower VL may have affected SFV detection using nested PCR of PBMC specimens (p = 0.038) but it did not affect the detection in buccal swab specimens (p = 0.669).

## Discussion

We developed novel qPCR assays to facilitate simultaneous detection and quantification of NWP SFV in clinical specimens, including noninvasively collected buccal swabs to overcome the limitations of small blood volumes typically obtained from small animals. Our validated real-time qPCR assay specifically detected and quantified highly divergent SFV from three families of neotropical primates with high sensitivity. Recently, another NWP SFV real-time PCR has been reported that was designed using *pol* sequences available at that time from only SFVmar, SFVsqu, and SFVspm complete genomes and optimized with a single SFVsqu sequence [14]. In that study, DNA VLs were only reported for two squirrel monkey blood specimens, having an average of 2.8 log SFV copies/million cells. These results are similar to those seen in PBMC DNA from three squirrel monkeys (average 2.1 log SFV copies/10^6^ cells) in our study, but lower than the average DNA VL (4.7 log SFV copies/10^6^ cells) found in buccal swab DNA from eight squirrel monkeys herein. Matching buccal swab specimens were not available from the two squirrel monkeys for qPCR testing in that previous study [14] for comparison with our findings. NWP SFV sequences were not detected in PBMC gDNA from all eight humans seropositive for NWP SFV antibodies in the Stenbak *et al*. study using their qPCR assay [14]. However, it is not clear if the limited validation reported for that qPCR assay and the likelihood of low DNA VLs may explain the negative PCR results in the seropositive primate workers.

We found no differences in SFV DNA VL among PBMCs collected from mature and immature NWPs and in male and female animals. Likewise, we did not find a correlation between age and DNA VL in two genera for which several specimens were available (*Sapajus* and *Leontopithecus*). These findings may suggest that DNA VLs in PBMC do not vary with sexual maturation or gender in NWP, and are not correlated with age for at least these two genera. This is congruent with the idea that SFV is latent in the PBMC compartment of infected animals. However, it is important to highlight the potential limitation of those analyses, as only 14 immature animals were available overall in the maturity analysis, and limited specimens of *Leontopithecus* (n = 12) were used in the age correlation assessment. Testing of larger numbers of specimens from these two genera and other species are required to unequivocally confirm these findings.

We also compared NWP SFV DNA VLs in PBMCs determined in our study with those reported for OWPs [25, 26]. Soliven *et al*. found a median DNA VL of 830 SFV copies/10^6^ cells (8.3 x 10^−4^ SFV copies/cell) in the blood of six rhesus macaques [25] and Murray *et al*. reported DNA VL levels ranging from 190 to 2,230 SFV copies/10^6^ cells in 11 SIV-negative rhesus macaques [26]. Combined, these results suggest that DNA VLs in the PBMC of SFV-infected NWP are similar to those seen in SFV-infected OWP.

Very little is known about DNA VLs in the oral mucosa in NWP and OWP since only one study measuring SFV DNA levels in the oral cavity of OWPs has been published [26]. That study found 500–100,000 SFV DNA copies/10^6^ cells in oral tissues (parotid salivary gland, tonsil and tongue) of 12 SIV-positive rhesus macaques [26] compared to a range of 142–20 million SFV copies/10^6^ cells in NWP buccal swabs in our study. While adequate specimen volume was not available to quantify SFV RNA copies in the NWP buccal swabs in our study and in that of Stenbak *et al*., two studies of OWPs found higher RNA levels than DNA levels in oral swabs, indicative of active viral expression [25, 26]. It is important to note the FVs incorporate both DNA and RNA in virus particles with DNA consisting of less than 50% of the particle-associated viral genomes [20]. Hence, detection of increased DNA in VL testing of buccal swabs may be consistent with identification of potentially infectious viral particles, which can be confirmed with virus tissue culture, but which was not done in our study for the limitations we already described. Alternatively, the higher DNA VLs seen in buccal swabs could also represent higher levels of infected cells in that compartment which could be clarified in subsequent studies.

When we compared SFV DNA VLs in buccal swabs and PBMC from the same monkeys we found that DNA VLs in buccal swabs were much higher (up to 3.9 log) than in PBMCs in agreement with SFV replication localized mainly in oral tissues [20]. These findings suggest that bites by SFV-infected NWPs may increase SFV transmission among NHPs and also to humans via bite exposures. Our results differ from those obtained from testing of 14 humans infected with gorilla SFV in which DNA VLs were about three times higher in PBMCs than in saliva [23]. However, saliva may dilute infected cells, compared to buccal swabs, decreasing SFV DNA concentration and the ability to accurately detect SFV in that specimen type. No differences in DNA VLs were observed between sexually mature and immature monkeys in both buccal swab and PBMC specimens in our study. Since SFV is typically latent in PBMC, we expected to find differences among mature and immature animals in buccal swabs following reports of viral load increases with age in the oral cavity in rhesus macaques [25]. However, our inability to see an age-related viral load trend in buccal swab samples may be that we have quantified DNA VL rather than RNA VL as reported in that previous study [25] and in part by the small number of sexually immature monkeys (n = 6) in our study.

We obtained some discordant results by nested and qPCR using both PBMC and buccal gDNA. Among the SFV-positive primates by nested PCR of PBMC and buccal specimens, 92% had detectable DNA VLs, and among those animals negative by nested PCR of PBMC and buccal gDNA, 50% had detectable DNA VLs, including nested PCR-negative animals testing positive by WB. We did not find any association between the discordance of both assays and the lack of specificity to detect SFV from specific species, as we did not find significant SFV detection rates among distinct SFV-infected NWP genera. Similarly, we did not find differences in qPCR positivity by gender or sexual maturity. Our results suggest however that the new qPCR assay has a much better sensitivity compared to nested PCR testing, especially using PBMC gDNA. This increased assay sensitivity is likely due to the use of multiple generic primers designed using a broader diversity of NWP SFV and detection of a shorter SFV *pol* sequence. This finding is supported by WB testing for 20 monkeys with negative nested PCR results but with detectable DNA VLs, for which the majority (18/20, 90%) were WB-positive. However, there were also a small number of WB-positive animals with qPCR-negative results mostly by testing of only PBMC gDNA. Testing of buccal gDNA may have improved detection in those animals belonging to the *Alouatta guariba* and *Chiropotes* species. In addition, the increased availability of SFV sequences may help improve the design of PCR primers to better detect SFV in those species and other NWPs not yet sampled. For example, primers for our original diagnostic nested PCR assay that detects short *pol* sequences were designed using the only three complete NWP SFV genomes that were available at that time [11]. Primer design for the qPCR assay used additional NWP *pol* sequences available at GenBank from a broader diversity of NWP SFV and was able to detect SFV in more NWPs most of which were also WB-positive, including two additional species of *Leontopithecus*, and one species each of *Callimico* and *Saguinus* previously found to be SFV-negative using the original nested PCR assay [11, 12]. Altogether, our findings suggest that the combined use of the novel qPCR assay described herein and WB testing will provide a more accurate estimate of SFV prevalence in NWPs.

In summary, we describe the first survey comparing SFV DNA VLs in PBMC and buccal swabs from highly diverse NWPs. We found higher DNA VLs in buccal swabs than PBMCs likely indicative of greater active replication in the oral cavity similar to that seen in OWPs, and suggesting that bites by SFV-infected NWPs may increase SFV transmission among NHPs and to persons with that specific exposure. We also provide new molecular tools for use in future NWP SFV studies *in natura* and in captivity. Our findings contribute to a better understanding SFV infection and epidemiology of this large primate group.

## Materials and methods

### NWP specimens and ethics statement

Buccal swab and blood samples were collected from captive NWPs in Brazil consisting of 13 genera across all three taxonomic families (*Atelidae*, *Cebidae*, and *Pitheciidae*), including *Alouatta*, *Aotus*, *Ateles*, *Brachyteles*, *Cacajao*, *Callimico*, *Callithrix*, *Cebus*, *Chiropotes*, *Leontopithecus*, *Saguinus*, *Saimiri* and *Sapajus*. NWPs were housed at CPRJ (n = 58) and at RIOZOO (n = 49). All samples were collected following the national guidelines and provisions of IBAMA (*Instituto Brasileiro do Meio Ambiente e dos Recursos Naturais Renováveis*, Brazil; permanent license number 11375–1), which included animal welfare standard operating procedures. The project was approved on 07/11/2014 by Ethics Committee on the Use of Animals (CEUA) of Universidade Federal do Rio de Janeiro with the reference number 037/14.

### Sample collection and processing

Animals were anesthetized for both venipuncture (blood) and buccal swab collection, which were performed simultaneously by veterinarians at each institution. One to five milliliters of whole blood was collected in EDTA-containing vacutainer tubes and samples were kept at 4°C and processed within 24 hours of collection with Ficoll-Paque™ Plus (GE Healthcare, USA) to separate PBMC and plasma which were then stored at -80°C. Buccal swabs were collected using sterile cotton swabs with a plastic shaft. The cotton swab was rubbed gently against the buccal cavity (tongue, palate and left and right sides of mucosa) and along the gingiva, saturating the swab with saliva. Swab samples were placed into 1.5 ml centrifuge tubes containing 500 μl of saline solution (0.9% NaCl) and the shaft of the swab was cut just above the cotton tip using flame-sterilized scissors before tubes were closed. Tubes were kept at -20°C until processing. gDNA was extracted from PBMC and buccal swab samples using the QIAGEN genomic DNA extraction kit (QIAGEN, Chatsworth, CA) according to the manufacturer’s specifications.

### Nested PCR

To confirm the integrity of the extracted gDNA, we PCR-amplified a 975-bp *cytB* mtDNA sequence from each specimen in our study using 100 ng PBMC gDNA and ~12 ng buccal swab gDNA as described previously [11]. All samples with detectable *cytB* sequences were considered suitable for SFV PCR detection.

To detect SFV viral DNA in NWPs, we first performed a screening PCR for short *pol* sequences (192-bp) using generic primers and 250–500 ng PBMC gDNA as previously described [11]. Primers were designed using the three complete genomes available during that study from marmoset, squirrel, and spider monkeys (accession numbers GU356395, GU356394, and EU010385, respectively). We refer to this assay as the diagnostic *pol* PCR test. In addition, generic primers were also used with 250–500 ng PBMC gDNA to amplify two additional SFV genomic regions, a 398-bp LTR/*gag*-sequence (225-bp in LTR and 173-bp in *gag*) and a 520-bp *pol* fragment, using nested PCR [11]. An animal was considered infected with SFV when specimens from the animal tested positive in any of the three PCR assays used in our study. For the diagnostic *pol* PCR assay, each sample was tested in triplicate in three different assay runs and a sample was considered positive if any of the replicates tested positive. This diagnostic *pol* PCR assay has a high sensitivity for detection of diverse NWP SFV variants with a reported 100% (92–100%, 95% confidence interval (CI)) sensitivity, and 91% specificity (79–97%, 95% CI) when compared with WB results [11, 12].

### Real-time qPCR development and validation

A real-time PCR assay was developed to quantify NWP SFV DNA copies present in PBMC and buccal swabs using the CFX96 Touch™ Real-Time PCR Detection System and the CFX Manager software (Bio-Rad Laboratories, Hercules, CA). Primers and probes were designed using an alignment of partial and complete NWP *pol* sequences generated in a previous study [11] and available at GenBank, respectively, including those from marmoset (*Callithrix jacchus* (GU356395), squirrel (*Saimiri* species (GU356394*)*), spider (*Ateles species* (EU010385)), howler (*Alouatta belzebul*, *A*. *guariba*, and *A*. *seniculus*), and capuchin (*Sapajas albifrons*, *S*. *apella*, and *S*. *xanthosternos*) monkeys. To validate the qPCR assay, partial SFV *pol* sequences from seven divergent NWP SFV strains (SFVcja, SFVsxa, SFVssp, SFVasp, SFVagu, SFVcme and SFVpsp, comprising viral strains from each NWP family (*Atelidae*, *Cebidae*, and *Pitheciidae*), were cloned into the TOPO^®^-TA cloning vector (Thermo-Fisher Scientific, Waltham, MA). The SFVcja and SFVssp plasmids were kindly provided by Joseph Sodroski. SFVasp was from viral isolate previously available at the CDC, that was cultured in Cf2Th cells and total genomic DNA has been extracted for PCR [12]. SFVpsp came from SFV-infected PBMC as described in [12]. The remaining three sequences came from SFV-positive PBMC samples described previously by our group [13]. Successful cloning of all PCR fragments was confirmed by DNA sequencing of the recombinant plasmids (data not shown). Ten-fold serial dilutions were prepared for each plasmid and absolute copy numbers for each plasmid were determined. Primers and probe were designed based on NWP SFV *pol* sequence alignments previously determined [11], including representative sequences from all three NWP families. The primers for this new assay are located within the region amplified for the diagnostic nested PCR assay. We used two forward primers (5’-TGC ATT CCG ATC AAG GAT CAG C-3’ and 5’-YTT TGC YRC TTG GGC MAM RGA VA-3’), one reverse primer (5’-TTC CTT TCC ACY WTY CCA CTA CT-3’), and one probe (5’-TGG GGI TGG TAA GGA G”T”A CTG WAT TCC A-3’) containing 5’-FAM as the reporter dye and BHQ1 as the quencher dye in a “T” configuration instead of at the 3’ end. Real-time PCR was performed using 1 μl of AmpliTaq Gold^®^ DNA polymerase (5 U/μl) (Thermo-Fisher Scientific), 5 μl 10x buffer, 3 μl MgCl_2_ (25 mM), 1 μl dNTP mix (10 mM), 0.5 μl each forward primer (20 mM), 1 μl reverse primer (20 mM), 1 μl probe (12.5 mM) and 100 ng gDNA in a 50 μl reaction volume. PCR conditions were 1 cycle of 95°C for 10 min, followed by 55 cycles of 95°C for 15 sec, 50°C for 30 sec and 62°C for 30 sec.

A generic real-time qPCR for the primate *RPP30* (ribonuclease P/MRP, 30kDa subunit) gene was developed based on an alignment of NWP *RPP30* gene sequences available in the GenBank database. For the *RPP30* qPCR test we used the forward and reverse primers (5’-GCA CAT TTG GAC CTG CGA G-3’ and 5’-TAG CCA AGG TGA GCG GCT G-3’, respectively) and the probe (5’-TTC CTT TCC ACY WTY CCA CTA CT-3’) containing 5’ HEX as the reporter dye and BHQ1 as the quencher dye at the 3’ end. The PCR-amplified *RPP30* sequence from a *Callithrix aurita* was cloned as described above. Real-time PCR was performed using 0.5 μl AmpliTaq Gold^®^ DNA polymerase (5 U/μl), 2.5 μl 10x buffer, 1.5 μl MgCl_2_ (25 mM), 0.5 μl dNTP mix (10 mM), 0.45 μl primers (20 mM), 1.25 μl probe (10 mM) and 1 μl PCR product in a 25 μl reaction volume with running conditions of a first cycle at 95°C for 10 min, followed by 55 cycles of 95°C for 15 sec, 55°C for 30 sec and 72°C for 30 sec. To quantify the number of input cells, a standard curve was generated using 10-fold serial dilutions of the RPP30 plasmid. Detection of two copies of *RPP30* was considered one cell equivalent. To express the SFV proviral load per cell equivalent, the ratio of SFV copies/cell equivalent was calculated for each sample and determined per million cells. All qPCR testing included negative DNA and water-only controls.

The dynamic linear range of the qPCR assays was determined using standard curves of 10-fold serial dilutions of each SFV (SFVcja and SFVasp) and *RPP30* plasmids from 10^8^ to 10^0^ copies/reaction. Assay sensitivity was measured using dilutions of the six SFV and single *RPP30* plasmids to 100, 50, 20, 10, 5 and 1 copy/reaction and by testing 10 replicates of each dilution. Specificity of the NWP SFV and *RPP30* qPCR assays was determined by testing of PBMC gDNA from 35 human blood donors from the U.S. previously testing negative for OWP and NWP SFV [12]. Additional specificity analysis of the SFV qPCR assay was done by testing of PBMC gDNA of 30 NWPs from 12 different species (*Aotus nigriceps*, *Ateles paniscus*, *Brachyteles arachnoides*, *Callicebus personatus*, *Callithrix jacchus*, *Leontopithecus chrysomelas*, *Leontopithecus chrysopygus*, *Sapajus robustus*, *Sapajus apella*, *Cacajao melanocephalus*, *Pithecia monachus* and *Saimiri sciureus*) previously shown to be SFV WB- and PCR-negative as described [13].

### Statistical analyses

All DNA VL data was tested for normality of distribution using the skewness and Kurtosis statistics, and all showed to have a normal distribution (data not shown). The Student’s *t* test was used to compare SFV DNA VLs in PBMC between mature and immature animals and between overall SFV DNA VLs in PBMC and buccal swabs. The Pearson correlation coefficient was used to evaluate possible associations between age and SFV DNA VLs. All statistical analyses were done using SPSS v.20 and Microsoft Excel for Windows.

### Serology

To clarify SFV infection status in animals with discordant nested and qPCR results we performed a WB assay using combined SFV antigens to screen for SFV antibodies in plasma from these monkeys [16]. To broadly detect NWP SFV, we used antigens from Cf2Th cell lysates infected with SFV from a common marmoset (*Callithrix jacchus*, SFVcja, ATCC VR-919) and a spider monkey (*Ateles* species, SFVasp) as described in detail previously [13]. Samples with seroreactivity to both p68 and p72 Gag precursor proteins with an absence of similar reactivity to antigens from uninfected Cf2Th cells were interpreted as seropositive. Specimens without reactivity to either Gag protein were considered seronegative [13].

## Supporting information

S1 Table Sexual maturity cutoffs used for the New World primate genera studied. (DOCX)

## References

[1] Kehl T, Tan J, Materniak M. Non-simian foamy viruses: molecular virology, tropism and prevalence and zoonotic/interspecies transmission. Viruses. 2013;5(9):2169–209. doi: 10.3390/v5092169 ; PubMed Central PMCID: PMC3798896. 24064793 10.3390/v5092169 PMC3798896

[2] Ayouba A, Duval L, Liegeois F, Ngin S, Ahuka-Mundeke S, Switzer WM, et al Nonhuman primate retroviruses from Cambodia: high simian foamy virus prevalence, identification of divergent STLV-1 strains and no evidence of SIV infection. Infect Genet Evol. 2013;18:325–34. doi: 10.1016/j.meegid.2013.04.015 . 23612320 10.1016/j.meegid.2013.04.015

[3] Leendertz SA, Junglen S, Hedemann C, Goffe A, Calvignac S, Boesch C, et al High prevalence, coinfection rate, and genetic diversity of retroviruses in wild red colobus monkeys (Piliocolobus badius badius) in Tai National Park, Cote d'Ivoire. Journal of virology. 2010;84(15):7427–36. doi: 10.1128/JVI.00697-10 ; PubMed Central PMCID: PMC2897606. 20484508 10.1128/JVI.00697-10 PMC2897606

[4] Huang F, Wang H, Jing S, Zeng W. Simian foamy virus prevalence in Macaca mulatta and zookeepers. AIDS Res Hum Retroviruses. 2012;28(6):591–3. doi: 10.1089/AID.2011.0305 . 22236106 10.1089/AID.2011.0305

[5] Hood S, Mitchell JL, Sethi M, Almond NM, Cutler KL, Rose NJ. Horizontal acquisition and a broad biodistribution typify simian foamy virus infection in a cohort of Macaca fascicularis. Virol J. 2013;10(1):326 doi: 10.1186/1743-422X-10-326 . 24180225 10.1186/1743-422X-10-326 PMC4228416

[6] Morozov VA, Leendertz FH, Junglen S, Boesch C, Pauli G, Ellerbrok H. Frequent foamy virus infection in free-living chimpanzees of the Tai National Park (Cote d'Ivoire). J Gen Virol. 2009;90(Pt 2):500–6. doi: 10.1099/vir.0.003939-0 . 19141461 10.1099/vir.0.003939-0

[7] Jones-Engel L, Steinkraus KA, Murray SM, Engel GA, Grant R, Aggimarangsee N, et al Sensitive assays for simian foamy viruses reveal a high prevalence of infection in commensal, free-ranging Asian monkeys. J Virol. 2007;81(14):7330–7. Epub 2007/05/04. JVI.00343-07 [pii] doi: 10.1128/JVI.00343-07 ; PubMed Central PMCID: PMC1933339. 17475645 10.1128/JVI.00343-07 PMC1933339

[8] Switzer WM, Tang S, Ahuka-Mundeke S, Shankar A, Hanson DL, Zheng H, et al Novel simian foamy virus infections from multiple monkey species in women from the Democratic Republic of Congo. Retrovirology. 2012;9:100 doi: 10.1186/1742-4690-9-100 ; PubMed Central PMCID: PMCPMC3524035. 23217108 10.1186/1742-4690-9-100 PMC3524035

[9] Mouinga-Ondeme A, Kazanji M. Simian foamy virus in non-human primates and cross-species transmission to humans in Gabon: an emerging zoonotic disease in central Africa? Viruses. 2013;5(6):1536–52. doi: 10.3390/v5061536 ; PubMed Central PMCID: PMCPMC3717720. 23783811 10.3390/v5061536 PMC3717720

[10] Switzer WM. Foamy Virus. 1 st ed Liu D, editor: CRC Press, Taylor & Francis Group; 2011 131–46 p.

[11] Muniz CP, Troncoso LL, Moreira MA, Soares EA, Pissinatti A, Bonvicino CR, et al Identification and characterization of highly divergent simian foamy viruses in a wide range of new world primates from Brazil. PLoS One. 2013;8(7):e67568 doi: 10.1371/journal.pone.0067568 ; PubMed Central PMCID: PMCPMC3701081. 23844033 10.1371/journal.pone.0067568 PMC3701081

[12] Ghersi BM, Jia H, Aiewsakun P, Katzourakis A, Mendoza P, Bausch DG, et al Wide distribution and ancient evolutionary history of simian foamy viruses in New World primates. Retrovirology. 2015;12:89 doi: 10.1186/s12977-015-0214-0 ; PubMed Central PMCID: PMCPMC4627628. 26514626 10.1186/s12977-015-0214-0 PMC4627628

[13] Muniz CP, Jia H, Shankar A, Troncoso LL, Augusto AM, Farias E, et al An expanded search for simian foamy viruses (SFV) in Brazilian New World primates identifies novel SFV lineages and host age-related infections. Retrovirology. 2015;12:94 doi: 10.1186/s12977-015-0217-x ; PubMed Central PMCID: PMCPMC4650395. 26576961 10.1186/s12977-015-0217-x PMC4650395

[14] Stenbak CR, Craig KL, Ivanov SB, Wang X, Soliven KC, Jackson DL, et al New World simian foamy virus infections in vivo and in vitro. J Virol. 2014;88(2):982–91. doi: 10.1128/JVI.03154-13 ; PubMed Central PMCID: PMC3911628. 24198412 10.1128/JVI.03154-13 PMC3911628

[15] Hussain AI, Shanmugam V, Bhullar VB, Beer BE, Vallet D, Gautier-Hion A, et al Screening for simian foamy virus infection by using a combined antigen Western blot assay: evidence for a wide distribution among Old World primates and identification of four new divergent viruses. Virology. 2003;309(2):248–57. . 12758172 10.1016/s0042-6822(03)00070-9

[16] Soliven K, Wang X, Small CT, Feeroz MM, Lee EG, Craig KL, et al Simian Foamy Virus Infection of Rhesus Macaques in Bangladesh: Relationship of latent proviruses and transcriptionally active viruses. Journal of virology. 2013 doi: 10.1128/JVI.01989-13 . 24109214 10.1128/JVI.01989-13 PMC3838251

[17] Pacheco B, Finzi A, McGee-Estrada K, Sodroski J. Species-specific inhibition of foamy viruses from South American monkeys by New World Monkey TRIM5{alpha} proteins. J Virol. 2010;84(8):4095–9. Epub 2010/02/05. JVI.02631-09 [pii] doi: 10.1128/JVI.02631-09 ; PubMed Central PMCID: PMC2849481. 20130055 10.1128/JVI.02631-09 PMC2849481

[18] Liu W, Worobey M, Li Y, Keele BF, Bibollet-Ruche F, Guo Y, et al Molecular ecology and natural history of simian foamy virus infection in wild-living chimpanzees. PLoS pathogens. 2008;4(7):e1000097 doi: 10.1371/journal.ppat.1000097 ; PubMed Central PMCID: PMC2435277. 18604273 10.1371/journal.ppat.1000097 PMC2435277

[19] Moreira MA, Bonvicino CR, Soares MA, Seuanez HN. Genetic diversity of neotropical primates: phylogeny, population genetics, and animal models for infectious diseases. Cytogenet Genome Res. 2010;128(1–3):88–98. doi: 10.1159/000291485 . 20389036 10.1159/000291485

[20] Murray SM, Picker LJ, Axthelm MK, Hudkins K, Alpers CE, Linial ML. Replication in a superficial epithelial cell niche explains the lack of pathogenicity of primate foamy virus infections. Journal of virology. 2008;82(12):5981–5. doi: 10.1128/JVI.00367-08 ; PubMed Central PMCID: PMCPMC2395144. 18400853 10.1128/JVI.00367-08 PMC2395144

[21] Rua R, Betsem E, Gessain A. Viral latency in blood and saliva of simian foamy virus-infected humans. PLoS One. 2013;8(10):e77072 doi: 10.1371/journal.pone.0077072 ; PubMed Central PMCID: PMC3792900. 24116202 10.1371/journal.pone.0077072 PMC3792900

[22] Smiley Evans T, Barry PA, Gilardi KV, Goldstein T, Deere JD, Fike J, et al Optimization of a Novel Non-invasive Oral Sampling Technique for Zoonotic Pathogen Surveillance in Nonhuman Primates. PLoS Negl Trop Dis. 2015;9(6):e0003813 doi: 10.1371/journal.pntd.0003813 ; PubMed Central PMCID: PMCPMC4457869. 26046911 10.1371/journal.pntd.0003813 PMC4457869

[23] Smiley Evans T, Gilardi KV, Barry PA, Ssebide BJ, Kinani JF, Nizeyimana F, et al Detection of viruses using discarded plants from wild mountain gorillas and golden monkeys. Am J Primatol. 2016;78(11):1222–34. doi: 10.1002/ajp.22576 . 27331804 10.1002/ajp.22576

[24] Auricchio P. Primatas do Brasil: Terra Brasilis Comércio de Material Didático e Editora; 1995.

[25] Soliven K, Wang X, Small CT, Feeroz MM, Lee EG, Craig KL, et al Simian foamy virus infection of rhesus macaques in Bangladesh: relationship of latent proviruses and transcriptionally active viruses. Journal of virology. 2013;87(24):13628–39. doi: 10.1128/JVI.01989-13 ; PubMed Central PMCID: PMC3838251. 24109214 10.1128/JVI.01989-13 PMC3838251

[26] Murray SM, Picker LJ, Axthelm MK, Linial ML. Expanded tissue targets for foamy virus replication with simian immunodeficiency virus-induced immunosuppression. Journal of virology. 2006;80(2):663–70. doi: 10.1128/JVI.80.2.663-670.2006 ; PubMed Central PMCID: PMC1346877. 16378969 10.1128/JVI.80.2.663-670.2006 PMC1346877

