# Supplementary material for: A non-invasive specimen collection method and a novel simian foamy virus (SFV) DNA quantification assay in New World primates reveal aspects of tissue tropism and improved SFV detection
Source: PLoS One. 2017 Sep 1;12(9):e0184251. doi: 10.1371/journal.pone.0184251 (PMC5581185; doi:10.1371/journal.pone.0184251)
Supplement: S1 Table — (DOCX) [file pone.0184251.s001.docx]

**Supplementary Table 1.** Sexual maturity cutoffs used for the New World primate genera studied.

| Primate Genus | Sexual maturity*  (age in months) |
| --- | --- |
| *Alouatta* | ≥ 60 female  ≥ 84 male |
| *Aotus* | ≥ 24 |
| *Ateles* | ≥ 48 – 60 |
| *Brachyteles* | ≥ 48 – 60 |
| *Cacajao* | ≥ 36 female  ≥ 72 male |
| *Callicebus* | ≥ 36 – 60 |
| *Callimico* | ≥ 14 |
| *Callithrix* | ≥ 14 – 18 |
| *Chiropotes* | ≥ 48 |
| *Leontopithecus* | ≥ 16 – 20 |
| *Pithecia* | ≥ 48 female  ≥ 84 male |
| *Saguinus* | ≥ 16 – 20 |
| *Saimiri* | ≥ 36 – 60 |
| *Sapajus* | ≥ 96 female  ≥ 48 male |

* As defined in [26] for each primate genus.
